# Development of Cryopreservation Techniques for Gorgonian (*Junceella juncea*) Oocytes through Vitrification

**DOI:** 10.1371/journal.pone.0123409

**Published:** 2015-05-26

**Authors:** Sujune Tsai, Wish Yen, Suchana Chavanich, Voranop Viyakarn, Chiahsin Lin

**Affiliations:** 1 Department of Biotechnology, Mingdao University, Peetow, Chang Hua, Taiwan; 2 Department of Post Modern Agriculture, Mingdao University, Peetow, Chang Hua, Taiwan; 3 Institute of Biochemistry & Environmental Science, University of Ottawa, Ottawa, ON, Canada; 4 National Museum of Marine Biology & Aquarium, Checheng, Pingtung, Taiwan; 5 Institute of Marine Biology, National Dong Hwa University, Checheng, Pingtung, Taiwan; 6 Reef Biology Research Group, Department of Marine Science, Faculty of Science, Chulalongkorn University, Bangkok, Thailand; Kansas State University, UNITED STATES

## Abstract

Gorgonian corals are slowly declining due to human interaction and environmental impacts. Cryopreservation of gorgonian corals is an *ex-situ* method of conservation, ensuring future reproduction. The present study assessed the vitrification properties of cryoprotectant (CPT) mixtures using the cryotop, cryoloop and open pulled straw (OPS) cryopereservation methods prior to experimentation on gorgonian (*Junceella juncea*) oocytes. Investigations of the equilibration and vitrification solutions’ (ES and VS) effect on oocytes throughout different incubation periods were conducted. The cryotop method was found to be the most successful in ensuring vitrification. The most favourable VS was composed of propylene glycol (PG), ethylene glycol (EG) and methanol with concentrations of 3.5M, 1.5M and 2M respectively. Experiments were performed using the cryotop method to cryopreserve *Junceella juncea* oocytes using VS2, the solution had the least impact on oocytes at 5°C rather than at 26°C. The success of the vitrification procedures was determined by adenosine triphosphate (ATP) levels in cooled-thaw oocytes and the highest viability obtained from the present study was 76.6 ± 6.2%. This study provides information regarding gorgonian corals’ tolerance and viability throughout vitrification to further advance the vitrification protocol on whip corals.

## Introduction

Gorgonian corals provide important habitats for many ecologically significant fish, as well as vital reef habitats for numerous invertebrates, including seahorse (*Hippocampus*) [1], oyster (*Pteria colymbus*) [2] and shrimp (*Neopontonides beaufortensi*) [3]. The growth and health of said corals rely on optimal environmental conditions. However, due to the ever-increasing detrimental effects of global warming, pollution, disease risks, and anthropogenic destruction, coral reefs are now facing significant threats [4]. As such, the conservation and preservation of coral systems should be made a priority.

Current cryopreservation techniques allow for *ex-situ* conservation of genetic diversity in biological materials, including DNA, gametes, somatic cells, embryos and tissues [5]. With regards to the cryopreservation of invertebrates, freezing techniques have been developed to successfully cryopreserve the eggs and embryos of oysters (*Crassostrea virginica*) [6], sea urchins (*Evechinus chloroticus*) [7], abalone (*Haliotis diversicolor supertexa*) [8], and polychaete worms (*Nereis virens*) [9]. However, as it stands now, there is no information available on the cryopreservation of coral oocytes and embryos. Factors limiting oocyte cryopreservation include their high chilling sensitivity, and low membrane permeability. Another factor is the large amount of yolk within, and their large size, resulting in a low surface area to volume ratio [10].

Vitrification is a promising cryopreservation technique which uses an ultra-rapid cooling rate and high concentrations of cryoprotectant (CPT) in the course of cooling. To date, there has been no report on the cryopreservation of oocytes and embryos in coral and other aquatic invertebrate species using vitrification. However, attempts at vitrification of embryos and oocytes have been conducted in fish species including zebrafish (*Danio rerio*) [11], common tench (*Tinca tinca*) [12], flounder (*Paralichthys olivceus*) [13,14] and turbot (*Scophthalmus maximus*) [15]. Although the viability of oocytes and embryos from those studies was evaluated only using staining markers (Trypan blue) and morphology (the chorion, perivitelline space size, blastoderm integrity and yolk opacity), they did demonstrate the potential for cryopreservation of fish oocytes and embryos using the vitrification method.

The focus of this study was on cryopreservation of oocytes of the coral species *Junceella juncea* through highly efficient vitrification methods. Due to the extremely high cooling rate produced by vitrification carrier systems, the injury from ice formation and hypothermic effect on the cell is reduced [16]. We have previously shown that coral oocytes were very sensitive to subzero temperatures and hard coral oocytes were more sensitive to chilling than gorgonian coral oocytes [17,18]. Therefore, vitrification may be a suitable technique to save coral populations as it bypasses the hyprothmic zone through direct contact of biological samples with liquid nitrogen.

## Results

### Cryo-devices

Vitrification properties of the single and combined CPTs were examined with different freezing carriers. The oocytes were loaded with a micropipette in the cryotop, cryolop and open pulled straw (OPS) and subsequently submerged in liquid nitrogen. Based on the results achieved throughout our experiments, cryotop was the most suitable carrier. Among many factors necessary for optimal vitrification, the most important included a fast cooling rate and CPTs with high viscosity and low volume. By using minimum capacity, cryotop (1μl) and cryolop (1μl) were able to achieve faster freezing than OPS (100μl) due to improved heat conductivity.

### Examination of glass-forming properties of individual CPTs

Three vitrification methods were used to determine glass-forming properties of CPTs, as indicated in Table 1. It was determined that the cryotop method is the most successful as it is able to produce vitrification of all the CPTs at the lowest concentrations. Various CPTs are capable of assisting the vitrification and preservation of cell function during vitrifying. The glass-forming properties of tested CPTs were in the order of dimethyl sulfoxide (DMSO) = Propylene glycol (PG) > Glycerol > Ethylene glycol (EG) > methanol. Therefore methanol required higher concentrations to ensure vitrification whilst there were no differences in glass-forming properties between DMSO and PG which required the lowest concentrations to achieve vitrification. The results from data gathered on different molarities of CPTs (Table 1), a combination of PG or glycerol was made with EG and methanol to obtain five VSs (Table 2).

**Table 1:** Minimum concentrations of CPAs required to undergo vitrification with the OPS, cryotop and cryoloop methods.

Vitrification method	Minimum concentration (M) of CPAs				
	Methanol	DMSO	PG	EG	Glycerol
OPS	9	4.5	4.5	6.5	5.5
Cryotop	8.5	4	4	6.5	4.5
Cryoloop	--	4.5	4.5	6	5

**Table 2:** VSs of PG and glycerol are synthesized with EG and methanol at different concentrations.

Solutions	Concentration of component (M)		
	PG/Glycerol*	EG	Methanol
VS1	4	1	2
VS2	3.5	1.5	2
VS3	3	2	2
VS4	2.5	2.5	2
VS5	2	3	2

* Ten solutions in total are formed using PG and glycerol separately, both contain the same concentrations.

### Examination of glass-forming properties of VSs

Glass forming properties of PG and glycerol-based VSs during cooling in the liquid nitrogen and warming in the original solution by 3 vitrification methods are given in Table 3. Although all the glycerol-based VS remained vitrified using the cryotop device on cooling, none of glycerol-based VS exhibited vitrification on warming. Only PG-based VS1 (composed of 4M of PG, 1M of EG and 2 M of methanol) was successful in achieving vitrification in every vitrification device during both cooling and warming. The cyotop and cryoloop methods were also able to achieve vitrification when PG-based VS2 was used. PG-based VS3 was found to vitrify in every vitrification device during cooling but was devitrified during warming.

**Table 3:** Glass forming properties of PG and glycerol VSs during cooling and warming by cryoloop, cryotop and OPS methods.

Solution	Original Solution		
	OPS	Cryotop	Cryoloop
Glycerol VS1	VD	VD	VD
Glycerol VS2	VD	VD	VD
Glycerol VS3	C	VD	C
Glycerol VS4	C	VD	C
Glycerol VS5	C	VD	C
PG VS1	VV	VV	VV
PG VS2	VD	VV	VV
PG VS3	VD	VD	VD
PG VS4	C	VD	C
PG VS5	C	VD	C

VV—indicates vitrification when cooled as well as when warmed. VD—indicates vitrification when cooled but devitrifies upon thawing. C—indicates crystallization occurs when placed in liquid nitrogen.

Table 4 showed glass forming properties of PG and glycerol-based VSs with 400μg/ml of anti-freeze protein (AFP) during cooling and warming using 3 vitrification methods. With the addition of AFP, PG and glycerol VS did not improve vitrification in the cooling and warming process. On the contrary, devitrification occurred in PG-based VS2 with AFP which displayed vitrification without AFP upon warming to 26°C.

**Table 4:** Glass forming properties of PG and glycerol VSs with 400μg/ml of AFP during cooling in the liquid nitrogen and warming in the original solution by cryoloop, cryotop and OPS methods.

Solution	Original Solution + 400μg/ml AFP		
	OPS	Cryotop	Cryoloop
Glycerol VS1	VD	VD	VD
Glycerol VS2	VD	VD	VD
Glycerol VS3	C	VD	C
Glycerol VS4	C	VD	C
Glycerol VS5	C	VD	C
PG VS1	VV	VV	VV
PG VS2	VD	VV	VD
PG VS3	VD	VD	VD
PG VS4	C	VD	C
PG VS5	C	VD	C

VV—indicates vitrification when cooled as well as when warmed. VD—indicates vitrification when cooled but devitrifies upon thawing. C—indicates crystallization occurs when placed in liquid nitrogen.

### Effects of PG-based ES and VS on oocytes

**Fig 1:**
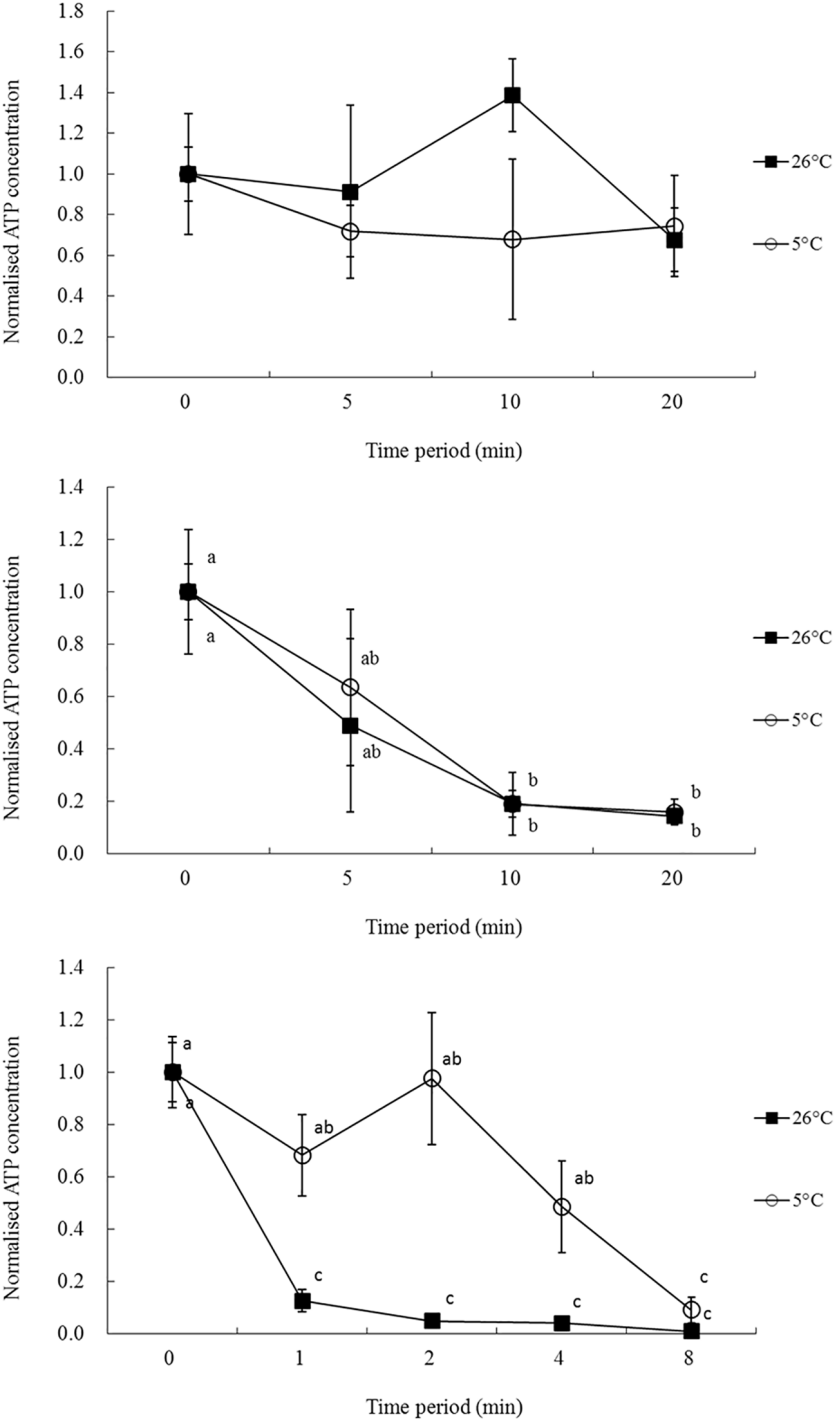
The effects of PG-based ES1 (a), ES2 (b) and VS2 (c) on *Junceella juncea* oocytes at 5 and 26°C for different time periods. Oocyte ATP levels were normalized to that of the control. Error bars represent standard errors. Different letters represent significant differences between temperature and exposure time period for oocytes treated with the same solutions (P < 0.05).

Normalized data on the effects of PG-based ES and VS at 5 and 26°C for different time periods are shown in Fig 1. When PG-based ES1 and ES2 were tested at 5 and 26°C (Fig 1a and 1b), there was no significant differences on adenosine triphosphate (ATP) level of oocytes after 5, 10 and 20 minute exposure (P > 0.05). As for the effect of different exposure period on oocytes, exposing oocytes to PG-based ES1 for up to 20 min demonstrated no effects on ATP level at both 5 and 26°C (P > 0.05). However, the amount of time at which oocyte ATP levels dropped significantly in PG-based ES2 was after 10 minutes exposure (P < 0.05). The effects of PG-based VS2 on oocytes was reduced when the PG-based VS2 was maintained at 5°C compared with 26°C (Fig 1c). Exposure of oocytes to PG-based VS2 at 26°C for more than 1 min was very toxic and the ATP level of oocytes decreased significantly compared with the control group (P < 0.05). Although the effect of PG-based VS2 was reduced at 5°C, a negative effect on oocytes started to appear after 4 min exposure (P < 0.05).

### Vitrification assays

**Fig 2:**
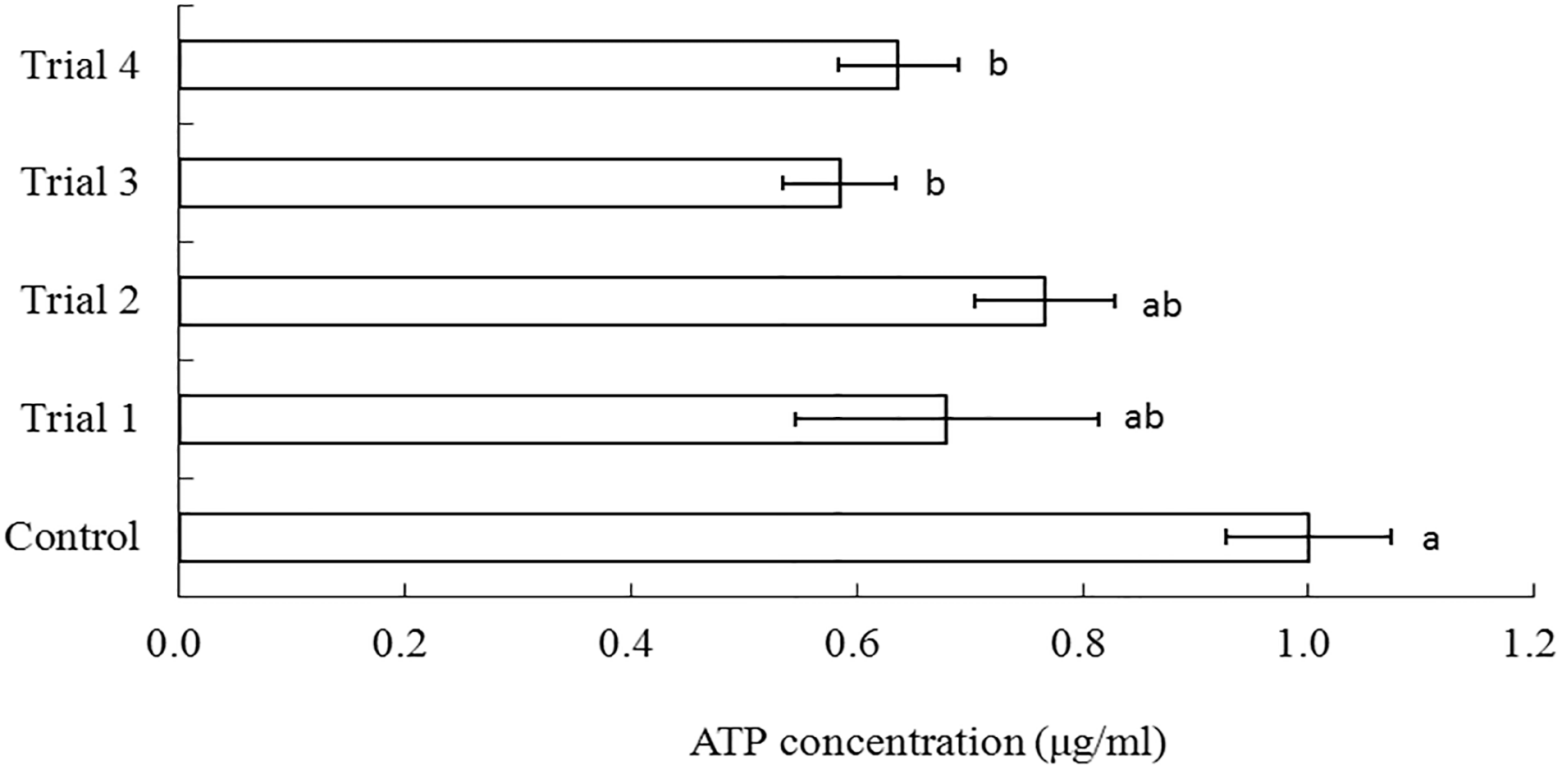
Normalized ATP concentrations of *Junceella juncea* oocytes after vitrification in PG-based VS2 for 2 min. Error bars represent standard errors. The bars with different letters are significantly different (P < 0.05). Trial 1: PG-based ES1 for 15mins, PG-based ES2 for 10mins; Trial 2: PG-based ES1 for 15 mins, PG-based ES2 for 5 mins; Trial 3: PG-based ES1 for 10mins, PG-based ES2 for 10mins; Trial 4: PG-based ES1 for 10mins, PG-based ES2 for 5mins.

The success of the vitrification procedures were determined by ATP levels in cooled-thaw oocytes. Cryopreservation was conducted using the cryotop device after oocytes were immersed in PG-based ES1 and ES2 together with PG-based VS2 at various time periods (Fig 2). Among the four trials, trial one produced a non-significantly different when compared with control. On the other hand, trial two, three and four demonstrated significantly lower ATP levels than control although oocytes from the trial two exhibited highest ATP level (76.6%).

## Discussion

We compared three vitrification devices (cryoloop, cryotop and OPS) to determine vitrification properties of selected CPTs in the first part of present study. The lowest concentration of all the CPTs to be vitrified was found with the use of the cryotop device. Due to higher thermal conductivity compared with other vitrification devices, cryotop has been reported to be highly efficient for the cryopreservation of human and bovine oocytes [19]. It is also possible that the minimum solution required to load on cryotop device allowed for an extremely rapid cooling rate [20]. A faster cooling rate is produced when low temperature transfer across devices with high thermal conductivity. Similarly, the small volume on the cryotop could also achieve extremely rapid warming rate which might avoid dreadful devitrification [21]. From the present study, the cryotop method was able to maintain vitrification of PG-based VS2 during warming. Thus, the cryotop device was chosen for the cryopreservation assay due to its superiority versus the cryoloop and OPS.

In order to determine utility of individual CPTs, equilibration at room temperature was attempted. CPTs were composed of DMSO, glycerol, PG, EG and methanol in various concentrations. The glass-forming properties of tested CPTs were found in the order of DMSO = PG > Glycerol > EG > methanol. This result is consistent with previous studies [11,22,23] which reported determination of glass-forming properties of CPTs following vitrification devices of nylon mesh, fiber plug and OPS. The results on different molarities of CPTs were then used to design vitrification solutions. DMSO was subsequently eliminated as it was determined to have significant negative effects on the oocytes from our previous study [24]. Despite requiring higher concentrations to ensure vitrification, methanol has been identified as a less harmful CPT [24,25], thus methanol was added to ensure oocyte survival.

Viscosity of the vitrification solution is one important factor in achieving vitrification. As vitrification is regarded as an extreme increase in viscosity, the protocol requires high concentrations of combined CPTs which are used to ensure that the oocytes are protected from crystallization throughout the protocol. Saragusty and Arav 2011 [26] reported that viscosity and the glass transition (Tg) temperature can be increased by using mixtures of cryoprotentants which also have lower impact than individual CPTs when compared on the same molar basis [16]. Indeed, currently aquatic oocyte and embryo vitrification protocols have been attempted with the use of a mixture of CPTs in zebrafish (*Danio rerio*) [11], common tench (*Tinca tinca*) [21], flounder (*Paralichthys olivceus*) [13,14] and turbot (*Scophthalmus maximus*) [15]and vitrification procedures have also been successful in mouse embryos [27] and vascular grafts [28]. In the present study, a total of ten combined cryoprotentant solutions were tested to determine their vitrification properties. Surprisingly, none of glycerol-based VS exhibited vitrification on warming and only PG-based VS1 and VS2 were able to achieve vitrification after warming. This may be related to physicochemical properties and hydrostatic pressure of the VSs [11]. Glycerol-based VS has lower Tg and intrinsically poor than PG-based VS therefore increasing the possibility of crystallization.

There are many supplements to prevent ice crystal formation and crystallization for vitrification procedure. Wowk et al. 2000 [29] demonstrated that adding synthetic polymer polyvinyl alcohol (PVA), a polymetic CPT inhibited the occurrence of devitrification in the glycerol and EG-based vitrification solution during warming. In the recent year, Vrana et al. 2011 [30] reported similar results that the ice inhibiting effects of carboxylated ε-poly-ι-lysine (PLL) was found in their study which successfully cryopreserved human induced pluripotent stem cells. Although the mechanisms of protective action on those synthetic polymers are still not clear, those authors developed a novel vitrification solution and opened new possibilities for controlling ice formation during cooling. Sugars such as sucrose and trehalose are the other supplements which can also help to vitrify the solutions. Sugars are commonly used as impermeable CPTs, often in combination with penetrating CPTs. Using sugars as ice inhibitors were effective on decreasing the cooling rate necessary to vitrify without crystallization [31]. AFP and Antifreeze glycoprotein (AFGP) have been reported as effective ice blockers for vitrification procedures [31,32]. They can attach to the outside of heterogeneous nucleators, in turn, the addition of further water molecules are stopped [33]. In the present study, addition of AFP did not improve vitrification in the warming process. We speculated that this result may be due to impurities of the AFP extract used and it was disappointing that the AFP used in this study was unable to maintain the vitrified state. However, seeking on-colligation ice blockers of devitrification stays attractive from cryobiologists who are researching on the properties of ice blockers.

To ensure oocyte survival in vitrification solutions prior to cryopreservation, it is necessary to add an ES to slowly transition oocyte membranes and ensure that osmotic pressure does not force the cell to hypotonicity. From our results, the oocytes exposed to PG-based VS2 for more than 1 min demonstrated negative effects at 26°C. The concentration of PG-based VS2 was too high for the oocytes to withstand. It is noticeable that oocytes only survived in PG-based VS2 for a minute. At 2 minutes and above, the oocyte began to suffer from hypotonicity and began to lyse. However, the effects of PG-based VS2 on oocytes were reduced when the PG-based VS2 was maintained at 5°C. It has been suggested that biochemical effect can be reduced by lowering exposure temperature [17,18]. Lower temperature excluded preferential co-solvent from binding to the protein therefore stabilized its native state without being denatured [34]. However, not all results from the present study support this hypothesis. For example, there were no significant differences in ATP level of oocytes after 5, 10 and 20 minute exposure when PG-based ES1 and ES2 were tested at 5 and 26°C. In some cases, lowering the temperature might compromise the permeability of CPTs. Indeed, *Drosophila* embryos took more than double the amount of time to return to its original volume after exposure to EG at 0°C and therefore lowering the temperature might cancel out the CPT protective action [35].

The final attempted cryopreservation protocol of *Junceella juncea* oocytes was developed with oocytes being submerged in PG-based VS2 using the cryotop prior to cooling in liquid nitrogen. Various experiments led to the elimination of the OPS, cryoloop as well as PG-based VS3 through VS5 in the protocol. The OPS and cryoloop were inefficient in retaining the vitrified solutions. PG-based VS3 through VS5 were eliminated due to the inability to vitrify when placed in liquid nitrogen. Among the four trials in the cryopreservation study, trial 1 produced a non-significantly different when compared with control. However, we did note that some oocytes appeared swelled and transparent. This may be indicating cooling injury by intracellular ice formation or osmotic shock which led on the fusion of yolk globule into one transparent mass [11].

Cryopreservation of gorgonian (*Junceella juncea*) oocytes through vitrification is reported here for the first time and showed highest viability of 76.6 ± 6.2%. Although the results obtained after vitification protocol using ATP assay were promising, several questions were raised by the present study related to problems of vitrification. Further studies aiming to answer the viability of the cooled-warmed oocytes after extended period of culture are now being carried out in our laboratory.

## Materials and Methods

**Fig 3:**
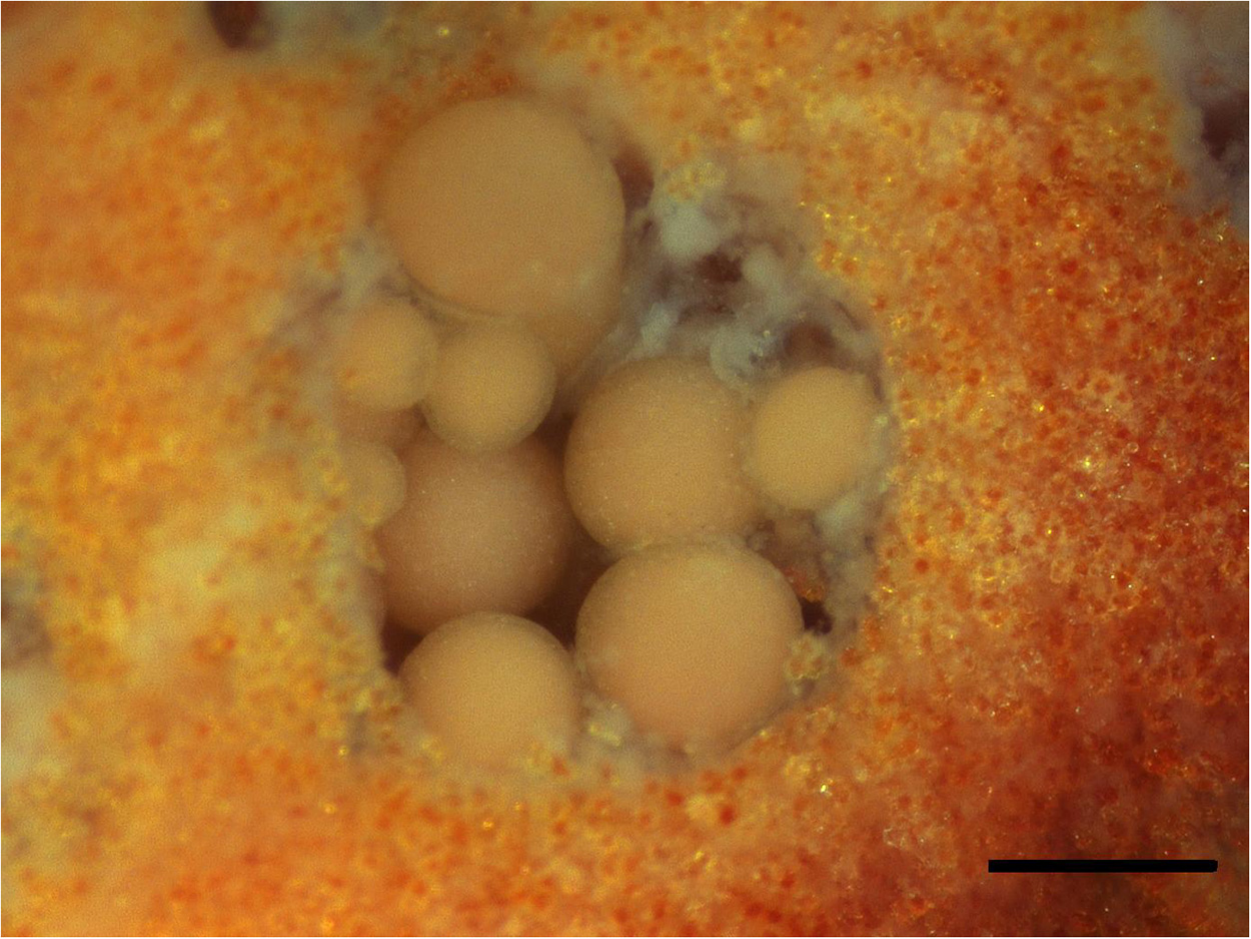
Biological and morphological characteristics of *Junceella juncea* oocytes observed using light microscopy. Scale bar = 300μm.

Ethics Statement: these corals are not regulated under Taiwanese law and the coral collection was approved by Kenting National Park. Coral Collection (21°56′N, 120°56′E) and oocyte isolation were following previously described [36,37]. The light microscopy (SZ51, Olympus, Japan) revealed that *Junceella juncea* oocytes were generally 50–300μm in diameter (Fig 3) and only the size of oocytes over 200 μm (late stage oocytes) were used in this study. Approximately 10 oocytes at different stages were found in a polyp cavity [38].

### Experimental design

**Fig 4:**
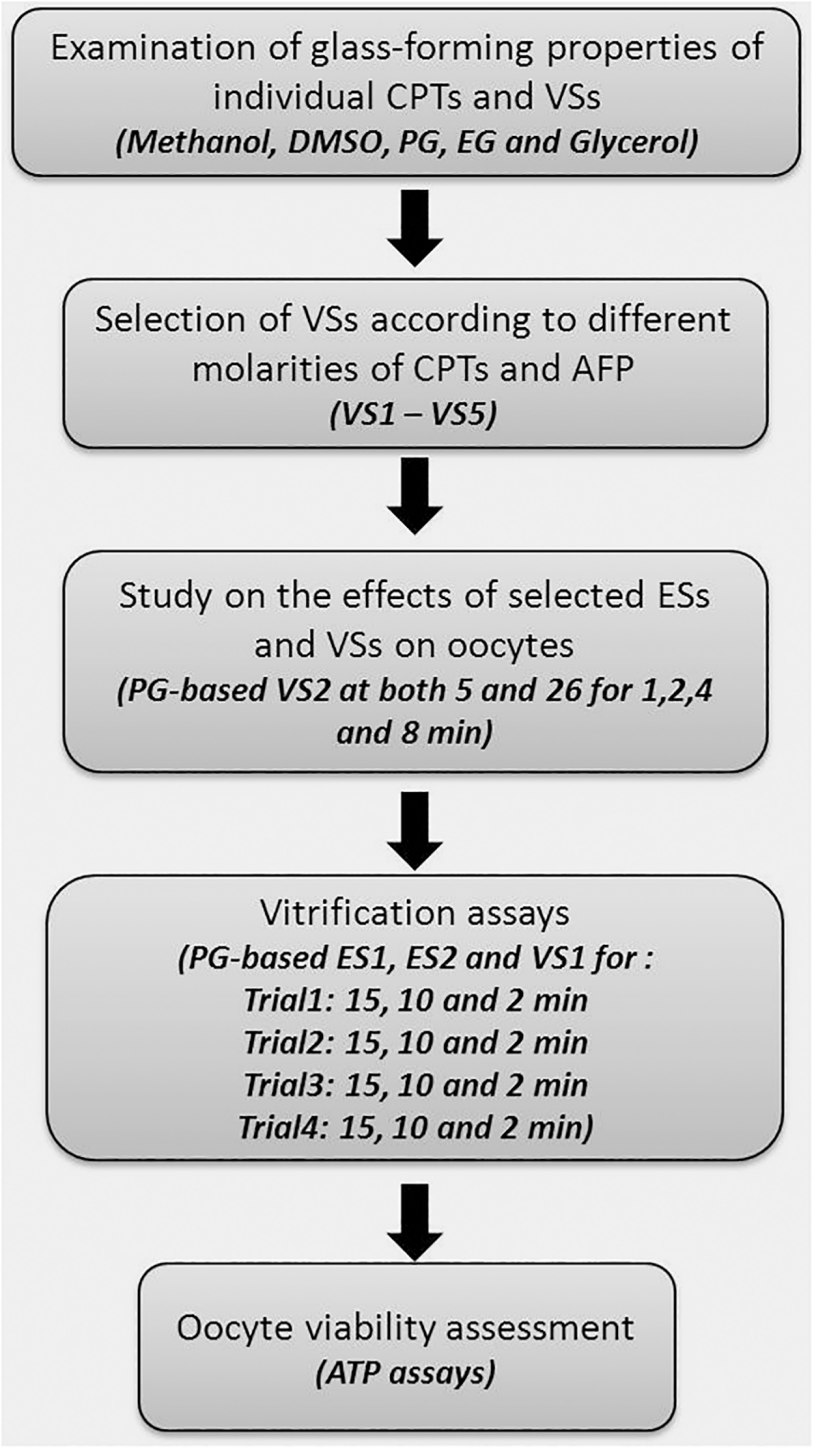
Flow chart of the experimental procedures.

The experimental procedures for this study were shown in Fig 4 including examination of glass forming properties of individual CPTs and VSs, selection of VSs according to different molarities of CPTs and AFP, study on the effects of selected ESs and VSs on oocytes, vitrification assays and oocyte viability assessment.

### Vitrification devices and CPTs

**Fig 5:**
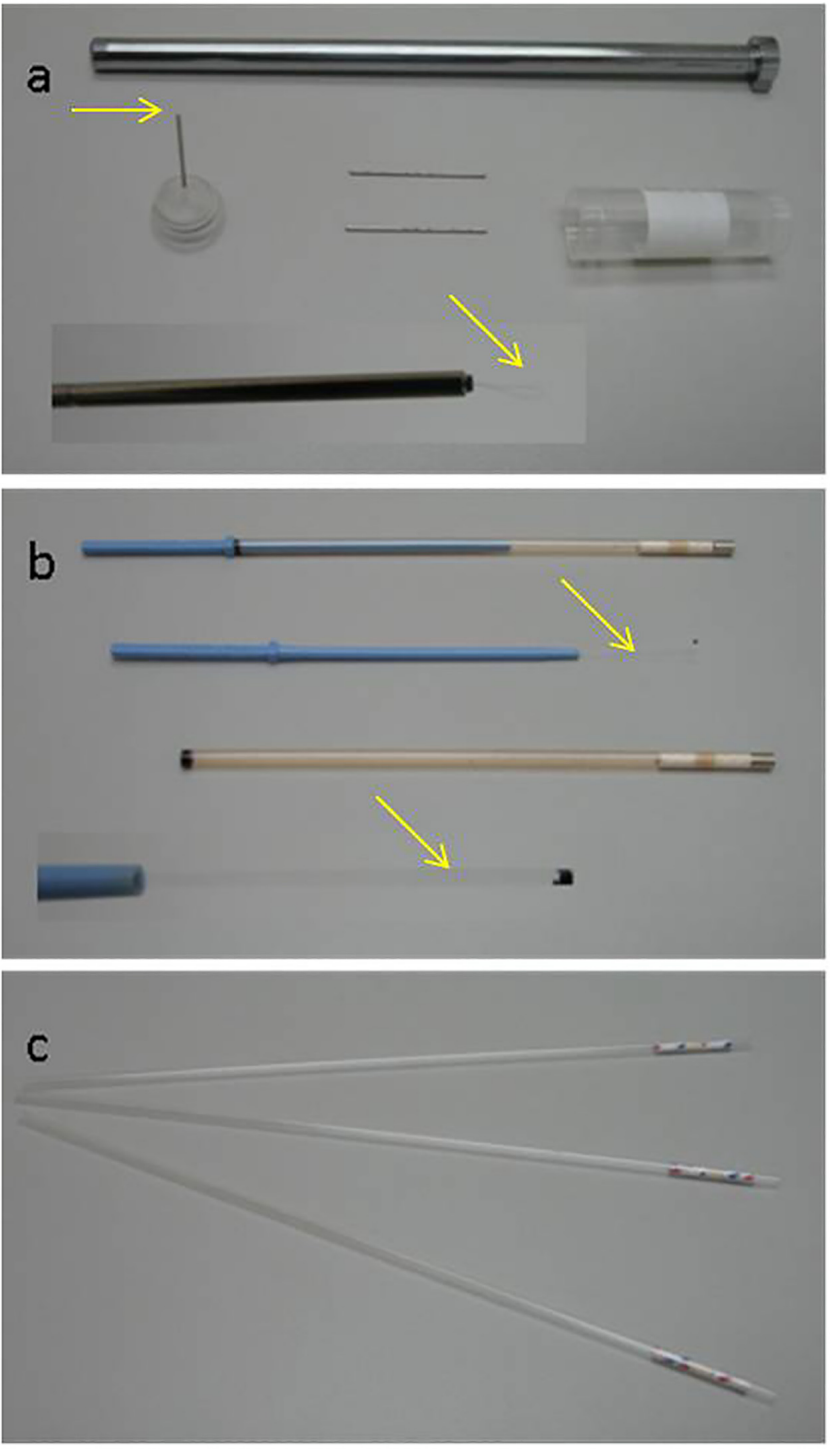
Vitrification devices used in present study: (a) Cryoloop, the oocytes were loaded on the loop area (arrows). (b) Cryotop, the oocytes were kept on the top of cryotop sheet (arrows). The black dot on the tip of the sheet is for ensuring the position of the oocytes when under liquid nitrogen. (c) OPS, the oocytes were aspirated in the straw by pipette tip.

Due to the ability to facilitate vitrification of minute amounts of oocytes, three specific devices: the cryoloop (Hampton research, USA), the cryotop (Kitazato, Japan) and OPS (imv Technologies, France) (Fig 5) were chosen to test whether vitrification would occur. Four CPTs including EG), PG, methanol, DMSO and glycerol as well as a biological CPT, AFP were used to create VSs in different combinations at specified concentrations. All chemicals were purchased from Sigma, St. Louis, MO USA.

### Examination of glass-forming properties of individual CPTs

Cryoloop, cryotop and OPS were used to determine vitrifying properties of EG (6.5–9 M), PG (4–6.5 M), methanol (8.5–10 M), DMSO (4.5–7 M) and glycerol (4.5–7 M) which were prepared in filtered sea water (0.45 μm filter) and underwent cooling in liquid nitrogen. Vitrification was considered successful when the CPT solution maintained its transparent appearance during cooling. The results from data gathered on different molarities of CPTs were then used to obtain VSs.

### Examination of glass-forming properties of VSs

The glass-forming properties of ten vitrification solutions (glycerol and PG-based VS1-VS5) (Table 2) were tested using OPS, cryotop and cryoloop methods as described above (glass-forming properties of individual CPTs). Another set of experiments was conducted by adding a 400 μg/ml AFP (from winter flounder; American Peptide, USA) to the same ten VSs. Warming was carried out by inserting vitrification devices into a 15 ml centrifuge tube which contained the original VS at 26°C. Non-vitrification or de-vitrification was considered when formation of ice crystals was visible in the VSs during cooling or warming.

### Effects of PG-based ES and VS on oocytes

The oocytes were placed in a six well culture dish (NUNC, Denmark) with 0.2μl filtered sea water. For experiments on the effect of PG-based ES, the oocytes were transferred into the other well containing PG-based ES1 (1M PG + 0.5M EG + 0.5M methanol) or ES2 (2M PG + 1M EG + 1M methanol) at both 5 (previously pre-chilled on ice) and 26°C for 5, 10 and 20 minutes. At the allotted time interval, 5 oocytes were removed and placed in a Petri dish filled with 0.2μl filtered sea water to wash the oocytes for three times. For experiments on the effect of PG-based VS, the oocytes were transferred into PG-based VS2 (3.5M PG + 1.5M EG + 2M methanol) at both 5 and 26°C for 1, 2, 4 and 8 minutes and followed the above produce to remove the VS. Mitochondria play a vital role in the energy metabolism processes in oocytes, providing ATP for the growth of embryos and the maturation of gametes. Throughout our coral research [39], we have found the concentration of ATP to be a direct corollary of metabolic activity, as well as a clear indicator of energy levels within oocytes. As such, the oocyte viabilities were assessed by determination of ATP levels using a luminescence assay (ApoSENSOR Cell Viability Assay Kit, BioVision, Cambridge BioScience, Cambridge, UK) in this study.

### Vitrification assays

Oocytes were placed in PG-based ES1, ES2 and VS1 for periods of 15, 10 and 2 minutes for trial one, 15, 5, and 2 minutes for trial two, 10, 10 and 2 minutes for trial three and 10, 5 and 2 minutes for trial four respectively. After serial equilibriums to PG-based ES and VS, oocytes were cooled using the cryotop method for 10 minutes in liquid nitrogen. Warming was carried out by immersing oocytes in PG-based VS 2 at 26°C for 2 min and then placed into ES2, ES1 and filtered sea water for the stepwise removal of CPT mixtures. The ATP level was then assessed as described above.

### Statistical analysis

The statistical analysis was computed using SPSS software (Version 17.0; SPSS Inc., Chicago, IL, USA). One-Sample Kolmogorov-Smirnov test and the Levene test were used to ensure the normality and homogeneity of variance of the data. A two-way ANOVA was then performed to test for the effects of treatments, time, temperature and their interaction on ATP level. Where there were differences, Tukey’s post-hoc test was carried out to locate differences. All data were presented as mean ± SEM across the three replicates and P values of less than 0.05 were considered to be significant.

## Supporting Information

S1 Table List of abbreviations. (TIF)
