# Supplementary figures and images for: Development of Cryopreservation Techniques for Gorgonian (Junceella juncea) Oocytes through Vitrification
Source: PLoS One. 2015 May 26;10(5):e0123409. doi: 10.1371/journal.pone.0123409 (PMC4444251; doi:10.1371/journal.pone.0123409)

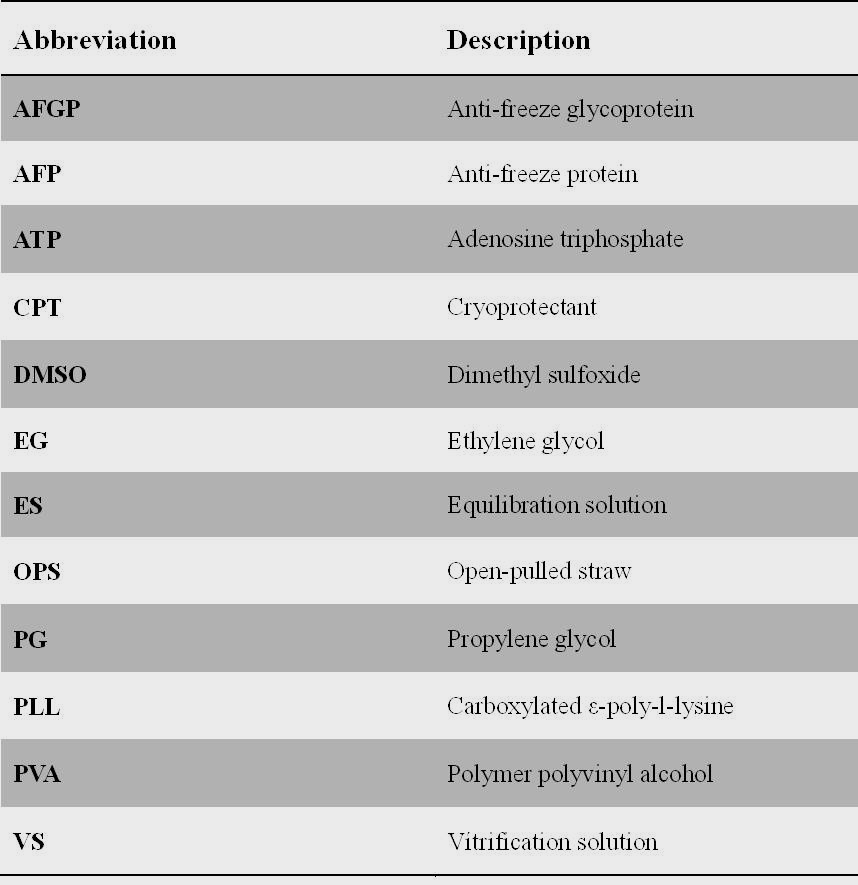

Supplement: S1 Table — (TIF) [file pone.0123409.s001.tif]
